# Possible Implications for Improved Osteogenesis? The Combination of Platelet-Rich Fibrin With Different Bone Substitute Materials

**DOI:** 10.3389/fbioe.2021.640053

**Published:** 2021-03-16

**Authors:** Sebastian Blatt, Daniel G. E. Thiem, Solomiya Kyyak, Andreas Pabst, Bilal Al-Nawas, Peer W. Kämmerer

**Affiliations:** ^1^Department of Oral and Maxillofacial Surgery, University Medical Center, Johannes Gutenberg University Mainz, Mainz, Germany; ^2^Platform for Biomaterial Research, BiomaTiCS Group, University Medical Center, Johannes Gutenberg University Mainz, Mainz, Germany; ^3^Department of Oral and Maxillofacial Surgery, Federal Armed Forces Hospital, Koblenz, Germany

**Keywords:** bone substitute, oral regeneration, platelet-rich fibrin, tissue engineering, osteoblast, allograft, xenograft

## Abstract

Bone substitute materials (BSM) are widely used in oral regeneration, but sufficient angiogenesis is crucial for osteogenesis. The combination of BSM with autologous thrombocyte concentrations such as platelet-rich fibrin (PRF) may represent a clinical approach to overcome this limitation. This study analyzes the early influence on osteoblast (HOB) *in vitro*. Here, four different BSM (allogeneic, alloplastic, and two of xenogeneic origin) were combined with PRF. After the incubation with osteoblasts for 24 h, cell viability, migration, and proliferation were assessed. Next, marker of proliferation, migration, and differentiation were evaluated on gene and protein levels in comparison to the native BSM and osteoblast alone. Addition of PRF increased viability for both the xenogeneic BSM (*p* = 0.0008, *p* = 0.032, respectively) in comparison to HOB and vs. native BSM (*p* = 0.008), and led to a tendency for increased cell proliferation and migration for all BSM (each *p* > 0.05). On gene basis, allogeneic and alloplastic BSM displayed a significantly increased RUNX2 expression (each *p* = 0.050). Expression of alkaline phosphatase for alloplastic (*p* = 0.050) and collagen-1 for xenogeneic BSM (*p* = 0.05) were significantly increased in combination with PRF. In addition, bone morphogenic protein was expressed significantly higher when xenogeneic material was combined with PRF in comparison to HOB alone (each *p* = 0.05). In summary, the combination of PRF with different BSM increases initial viability and may influence early proliferation and migration potential of osteoblast via RUNX2, alkaline phosphatase, collagen, and BMP2 especially in combination with alloplastic and xenogeneic BSM. Biofunctionalization of BSM using PRF might improve osteogenesis and extend the range of indications.

## Introduction

Autologous bone augmentation remains the treatment therapy of choice for regenerative craniomaxillofacial surgery in case of facial bone loss due to trauma, cancer, or other pathologies (Tatullo et al., 2012). However, disadvantages may be seen in the limited offer and enhanced morbidity with respect to the donor site especially in multimorbid patients (von Arx et al., 2001). Here, bone substitute materials (BSM) of allogeneic, xenogeneic, or alloplastic origins represent a suitable and promising therapy option with specific indications: in opposite to the osteoinductive capacity of autologous bone, BSM shows functional deficits due to their osteoconductive properties (Khosropanah et al., 2018). Only for allogeneic BSM, an osteoinductive potential could be demonstrated (Miron et al., 2016). Therefore, allogeneic materials in particular are frequently used for “bone engineering” where, e.g., via co-culture experiments, stem cell therapy or the addition of growth factors BSM were edited in order to improve bone regeneration procedures (Hinze et al., 2010).

As a key role in initial osteogenesis, a sufficient blood vessel supply and angiogenesis, the formation of new blood vessels from existing lumina, is mandatory (Rather et al., 2019). On the one hand, capillary structures supply the regenerated bony defect area with nutrients and minerals for homeostasis. In addition, they support and regulate diverse functions of the bone marrow and bone in osteogenesis processes, structurally and via paracrine pathways on different cellular levels (Grosso et al., 2017). Here, new engineering strategies may overcome the current limitations of an insufficient initial blood supply of BSM that, with an increased angiogenic potential, may lead the way to an optimized osseous regeneration.

Autologous platelet concentrate (PC) such as platelet-rich fibrin (PRF) are now broadly used in dental and craniomaxillofacial regenerative medicine (Dohan et al., 2006). Via the complex interplay of different cytokines and growth factors, the proliferation and differentiation of different cell lines is thrived (Miron et al., 2017). So far, a significant pro-angiogenic effect of the PRF could be shown especially for soft tissue regeneration procedures (Ghanaati et al., 2018; Blatt et al., 2020). Up to date, there is inconsistent data if PRF may also support bony regeneration (Miron et al., 2017). Still, raising evidence emerges that PRF may also support differentiation and proliferation of osteoblasts (Dohle et al., 2018). Lately, our working group demonstrated a positive effect after 3–10 days of co-incubation, especially in combination with an allograft in comparison to BSM alone or in combination with xenogeneic materials (Kyyak et al., 2020). However, data for a possible initial and early interaction remain spares.

Controversially, some studies and case reports report conflicting data if PRF may influence osteogenesis (Pripatnanont et al., 2013; Yoon et al., 2014). A possible explanation for the ambivalent data may be seen in the diversity of the analyzed BSM and their different biophysical properties. Furthermore, different time points of evaluation were chosen that counteract time points of the physiological wound healing phase. Therefore, the aim of this study was to investigate the early effect on viability, migration, proliferation, and differentiation of osteoblasts of the PRF when combined with BSM *in vitro* after 24 h. This way, a comprehensive understanding of the possible initial mechanism of PRF in comparison to the well-studied later time points in osteogenesis should be provided to detect intercellular implications and provide basic scientific evidence for potential clinical translation.

## Materials and Methods

### Bone Substitute Materials

Four commercially available BSM were tested: allogeneic (AKM: maxgràft^®^, botiss biomaterials GmbH, Zossen, Germany, granularity <2 mm), alloplastic (APKM: maxresob^®^, botiss biomaterials GmbH, Zossen, Germany, granularity 0.8–1.5 mm), and xenogeneic BSM (XKM1: cerabone^®^, botiss biomaterials GmbH, Zossen, Germany, granularity 1.0–2.0 mm, XKM2: BioOss^®^, Geistlich Pharma AG, Wolhusen, Switzerland, granularity 1−2 mm) were used for the further experiments.

### PRF Protocol

For the PRF protocol, blood was collected from three healthy volunteers who gave their informed consent to this study in accordance with the ethical standards of the National Research Committee (Ärztekammer Rheinland-Pfalz, no. “2019-14705_1”) and the 1964 Helsinki declaration and its later amendments or comparable ethical standards. Ten milliliters of peripheral venous blood per sample were collected after puncturing the cephalic or the median cubital vein with the vacutainer system and specific sterile plain vacuum tubes with additional silicone within their coating surface for solid (A-PRF+, Mectron, Carasco, Italy) and liquid PRF, respectively (iPRF, Mectron, Carasco, Italy). Next, PRF was directly manufactured (1,200 rpm for 8 min, relative centrifugal force 177 g at a fixed angle rotor with a radius of 110 mm, Duo centrifuge, Mectron, Carasco, Italy), as previously described (Blatt et al., 2020).

### Cell Culture

Before the incubation with osteoblast, PRF was pressed to a stable membrane with the “PRF Box” (Mectron, Carasco, Italy) as indicated by the manufacturer. Next, PRF was cut into small pieces of 10–20 mm^2^, 0.3–0.5 ml of liquid PRF was added and mixed manually with an equal quantity of the respective BSM (100 mg) to obtain a sticky clot. Next, a commercially available human osteoblast cell line (HOB, PromoCell, Heidelberg, Germany) was used and cultivated with a standard HOB medium with an additive fetal calf serum (FCS, Gibco Invitrogen, Karlsruhe, Germany), Dulbecco’s modified Eagle’s medium (DMEM, Gibco Invitrogen), dexamethasone (100 nmol/l, Serva Bioproducts, Heidelberg, Germany), L-glutamine (Gibco Invitrogen), and streptomycin (100 mg/ml, Gibco Invitrogen). Cultivation was done at 37°C in a constant, humidified atmosphere with 95% room air and 5% CO_2_ until a confluence of approximately 70% was reached. Next, HOB were passaged using 0.25% trypsin (Seromed Biochrom KG, Berlin, Germany). HOB at passage five were used and seeded in a 24 well plate (Merck, Darmstadt, Germany) in a density of 5 × 10^4^ cells per well. Now, 100 mg of the respective BSM were added in combination with (prepared as mentioned above) or without PRF and further incubated for 24 h at 37°C with 95% room air and 5% CO_2_. HOB alone served as control.

### Cell Viability Analysis

Next, cell viability was analyzed after 24 h by 3-(4,5-dimethylthiazol-2-yl)-2,5-diphenyltetrazolium bromide (MTT) assay, as previously described (Pabst et al., 2015). In brief, MTT (200 μL, 2 mg/mL) was added to the wells and incubated for 4 h at 37°C before the culture medium was discarded, and 10 ml of lysis buffer was added per well. Finally, a fluorescence microplate reader (Versamax, Molecular Devices, San Jose, CA, United States) was used at 570 nm to detect metabolic activity that reflects viability.

### Cell Proliferation Analysis

Fluorescence red was applied after 24 h with CellTracker (Life Technologies, Thermo Fisher Scientific, Darmstadt, Germany) according to the manufacturer’s instructions to track cell number and therefore, proliferation rate. After the removal of the culture media, warmed Red dye was added and incubated for 30 min. Afterward, the dye was removed, washed with serum-free medium, and incubated for 30 min. Finally, Red fluorescence was analyzed with a fluorescence microscope (BZ-9000, Keyence, Osaka, Japan). Automatic thresholding was applied to extract cell structures and the area fraction (%) was calculated as previously described (Kyyak et al., 2020).

### Cell Migration Assay

A scratch test was used to detect migration ability, as previously described (Kyyak et al., 2020). HOB were incubated with BSM in combination with and without PRF in a special scratch assay plate (ibidi GmbH, Gräfelfing, Germany) for 24 h at the above mentioned conditions. Here, red cell tracker was applied as mentioned above. Quantification of the migrated cells and visualization of cell viability was done with the ImageJ software (ACTREC, Navi Mumbai, India), as previously described (Kyyak et al., 2020). In brief, images at a 10× fold magnification were first converted to grayscale before image subtraction was used to correct background staining. Next, automatic thresholding was applied to extract cell structures, and cells migrated in the gap were evaluated and the area fraction (%) was calculated.

### ELISA Quantification

Growth factor release on protein basis was analyzed after co-incubation with 1.4 ml of the cell supernatant, which was extracted after incubation for 24 h with HOB and the respective native and bio-activated BSM samples, as previously described (Blatt et al., 2020). Antibodies for alkaline phosphatase (AP), collagen (COL), bone morphogenic protein 2 (BMP), osteocalcin (OCN), and Runt-related transcription factor-2 (RUNX, all R&D Systems, Minneapolis, MN, United States) were evaluated according to the manufacturer’s protocol and analyzed via an ELISA plate reader and the specific software (SoftMax Pro 5.4, Molecular Devices, San Jose, CA, United States). In brief, after diluting the capture antibody in a coating buffer according to the manufacturer’s dilution protocol, a 96-well-plate was coated with 100 μL per well of coating solution and incubated overnight at 2–8°C. Afterward, wells were washed with a wash buffer and the excess liquid was removed. Two hundred microliters of blocking buffer was added and incubated for 1 h at room temperature and then removed. Next, 100 μl of standards and samples were added into the designated wells and incubated for 1 h at room temperature. The sample was then aspirated, the plate was then washed three times, and the excess liquid was removed. According to the manufacturer’s instructions, detection body was diluted in the blocking buffer and 100 μl was added to each well. After incubating for 2 h at room temperature, the plate was washed and the excess liquid was removed. Next, 100 μl of streptavidin-HRP diluted in the blocking buffer was added and incubated for 30 min at room temperature. After washing and removing the excess liquid, 100 μl of TMB substrate solution was added to each well and incubated for 30 min, then, 100 μl of stop solution was added and absorbance at 450 nm was measured with the ELISA plate reader and the specific software.

### PCR Quantification

The evaluation of proliferation and migration marker on gene basis were done with real-time quantitative PCR (qRT-PCR, CFX Connect Real-Time PCR Detection System, Bio-Rad, Germany) using SYBR Green Supermix (BioRad, Hercules, CA, United States), as previously described (Kyyak et al., 2020), for the following genes: alkaline phosphatase (*ALPL*), bone morphogenic protein 2 (*BMP2*), collagen type 1 alpha 1 chain (*COL1A1*), bone gamma-carboxyglutamate protein (alias: osteocalcin, *OCN*), and RUNX family transcription factor 2 (*RUNX2*). For internal control, housekeeping genes actin alpha 1, skeletal muscle (*ACTA1*), and glyceraldehyde-3-phosphate dehydrogenase (*GAPDH*) were ran (primer sequences: Table 1). Briefly, the total RNA was extracted after 24 h of co-incubation using a commercial kit (Qiagen, Hilden, Germany) before RNA was converted to cDNA by the iScript cDNA synthesis kit (BioRad, Hercules, CA, United States) according to the manufacturers’ instructions. Eleven microliters of SYBR, 1 μl of primer sense, 1 μl of primer antisense, and 5 μl of RNA-free water were used with the thermal cycler at the first step −95°C for 3 min; second Step (repeated 39 times) −95°C for 10 s, then 58°C for 30 s, and finally 72°C for 20 s; final step −65°C for 0.5 s and then 95°C for 5 s. Quantification of gene expression was evaluated via the ΔΔCT method.

**TABLE 1:** Primer sequences for PCR protocol.

Primer	Sequence
*ACTA1*	Sense-GGAGCAATGATCTTGATCTT, antisense-CTTCCTGGGCATGGAGTCCT
*GAPDH*	Sense-AAAACCCTGCCAATTATGAT, antisense-CAGTGAGGGTCTCTCTCTTC
*ALPL*	Sense-ACTGCAGACATTCTCAAAGC, antisense-GAGTGAGTGAGTGAGCAAGG
*BMP2*	Sense-CCTGAAACAGAGACCCACCC; antisense-TCTGGTCACGGGGAATTTCG
*COL1A1*	Sense-AGAACTGGTGCAAG; antisense-GAGTTTACAAGACA
*OCN*	Sense-GSAAAGGTGCAGCCTTTGGT; antisense-GGCTCCCAGCCATTGATACAG
*RUNX2*	Sense-CCCACGAATGCACTATTCC; antisense-GGACATACCGAGGGACAT

### Statistical Analysis

The results were interpreted in mean values with its standard error and rounded to the first decimal place. For normal distribution, the Shapiro–Wilk test was used. In case of normally distributed values, the Student’s *t*-test for paired samples was applied. For non-normal distributions, the Mann–Whitney test was used. In order to compare all the groups, the Kruskal–Wallis rank sum test was applied. A *p*-value of ≤0.05 was considered to be statistically significant. Finally, bar charts with error bars were used for data illustration.

## Results

### Combination of PRF With BSM Increases Initial Viability and Tent to Improve Early Proliferation and Migration Potential of Osteoblast

**FIGURE 1:**
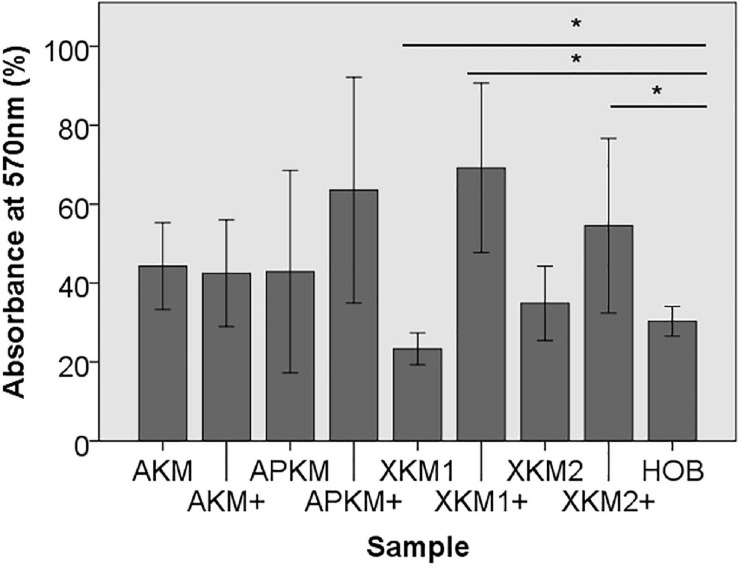
MTT assay to evaluate viability at absorbance of 570 nm of HOB after co-incubation with the respective samples with (+)/without PRF (*p < 0.05 Mann–Whitney *U* testing vs. HOB, XKM1: *p* = 0.016, XKM1+: *p* = 0.008, XKM2+: *p* = 0.032).

First, viability of HOB after 24 h of incubation with the respective BSM with or without PRF was analyzed via 3-(4,5-dimethyl-2-yl)-2,5-diphenyltetrazolium bromide (MTT) assay (five samples in triplets each, *n* = 60, Table 2A). Here, all samples leveled over the negative control of HOB except for XKM1. There was no statistical significance between the groups (*p* = 0.467). In comparison to HOB alone, xenogeneic BSM did reveal a statistically significant increased viability (XKM1: *p* = 0.016, XKM1+: *p* = 0.008, XKM2+: *p* = 0.032 all other tested samples: *p* > 0.05). Metabolic activity was significantly higher for xenogeneic material 1 only when combined with PRF vs. the native material (^∗^*p* = 0.008, all other tested samples: *p* > 0.05, Figure 1). The differences between the groups were statistically significant (*p* = 0.007, Figure 1).

**TABLE 2:** **(A)** MTT assay: Mean absorbances found at 570 nm for the respective samples with (+)/without PRF and respective *p*-values vs. HOB alone and native BSM, **(B)** Cell proliferation assessed with cell tracker red: Mean number of cells with its standard error and respective *p*-values vs. HOB alone and native BSM, **(C)** Scratch assay: Mean number of cells migrated into the gap for the respective samples with (+)/without PRF and respective *p*-values vs. HOB alone and native BSM.

(A)			
**Sample**	**Mean absorbance at 570 nm**	***p*-Value (respective sample vs. HOB, Mann–Whitney *U* test)**	***p*-Value (respective sample with PRF vs. native control, Mann–Whitney *U* test)**

HOB	0.303 ± 0.03	–	–
AKM	0.443 ± 0.11	0.095	0.690
AKM+	0.425 ± 0.13	0.151	
APKM	0.429 ± 0.25	0.310	0.412
APKM+	0.635 ± 0.28	0.151	
XKM1	0.232 ± 0.04	0.016	0.008
XKM1+	0.698 ± 0.21	0.008	
XKM2	0.348 ± 0.09	0.222	0.222
XKM2+	0.545 ± 0.22	0.032	

**(B)**			

**Sample**	**Mean number of cells**	***p*-Value (respective sample vs. HOB, Mann–Whitney *U*-test)**	***p*-Value (respective sample with PRF vs. native control, Mann–Whitney *U* Test)**

HOB	4.843 ± 8.906	–	–
AKM	5.627 ± 12.963	0.439	0.121
AKM+	5.291 ± 6.771	0.121	
APKM	4.228 ± 10.464	0.439	0.439
APKM+	5.403 ± 6.893	1.00	
XKM1	4.970 ± 11.287	0.439	0.121
XKM1+	5.239 ± 5.694	0.121	
XKM2	2.197 ± 6.181	0.121	1.00
XKM2+	4.175 ± 5.746	0.121	

**(C)**			

**Sample**	**Mean number of cells migrated into the gap**	***p*-Value (respective sample vs. HOB, Mann–Whitney *U* test)**	***p*-Value (respective sample with PRF vs. native control, Mann–Whitney *U* test)**

HOB	3.584 ± 5.40	–	–
AKM	3.834 ± 5.688	0.439	1.00
AKM+	6.878 ± 7.634	0.439	
APKM	6.230 ± 6.33	1.00	0.121
APKM+	5.236 ± 9.12	0.439	
XKM1	5.813 ± 6.757	0.439	0.439
XKM1+	6.769 ± 9.368	0.439	
XKM2	3.116 ± 4.537	1.00	0.439
XKM2+	4.872 ± 5.94	1.00	

**FIGURE 2:**
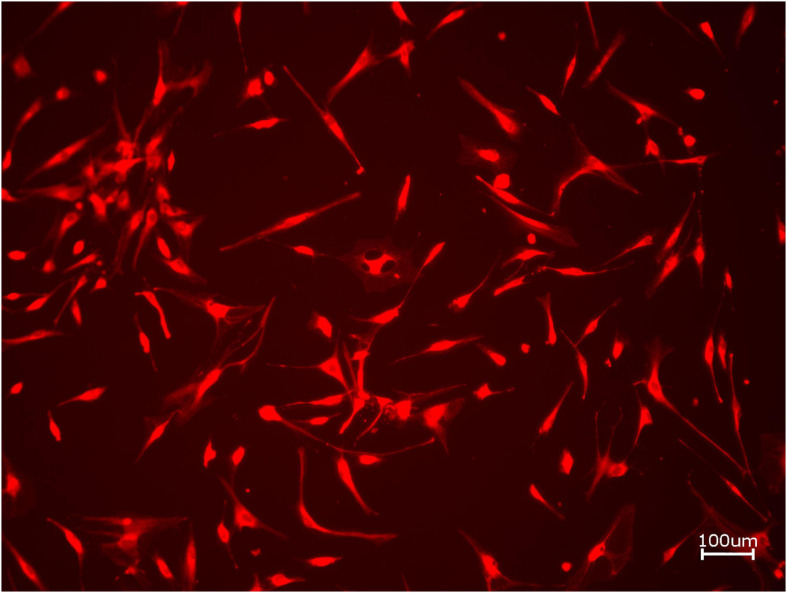
Exemplary micrograph of cell tracker red for allogeneic BSM without the addition of PRF (10× magnification).

**FIGURE 3:**
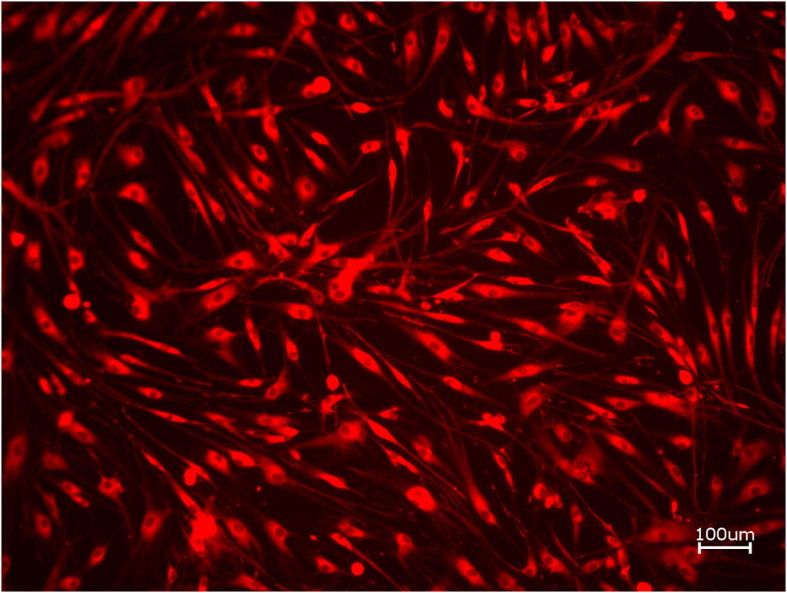
Exemplary micrograph of cell tracker red for allogeneic BSM with the addition of PRF (10× magnification).

**FIGURE 4:**
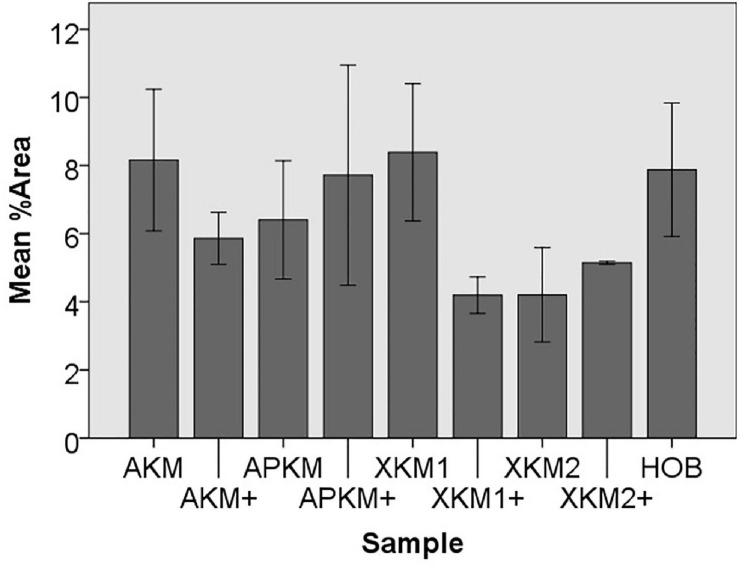
Mean percentage of cells assessed via cell tracker assay of HOB after co-incubation with the respective samples with (+)/without PRF to detect proliferation potential (each *p* > 0.05, Mann–Whitney *U* testing in comparison to HOB and native BSM).

Next, cell proliferation was investigated via cell tracker (Table 2B, three samples in duplets for each, *n* = 54). After 24 h of incubation, no significant differences between the groups could be revealed (*p* = 0.098). However, the addition of PRF led to a tendency for increased viability for APKM in comparison to HOB (all tested samples: *p* > 0.05). Viability was increased when alloplastic and xenogeneic materials where combined with PRF in comparison to their native control, however without reaching statistical significance (all tested samples: *p* > 0.05, Figures 2–4).

**FIGURE 5:**
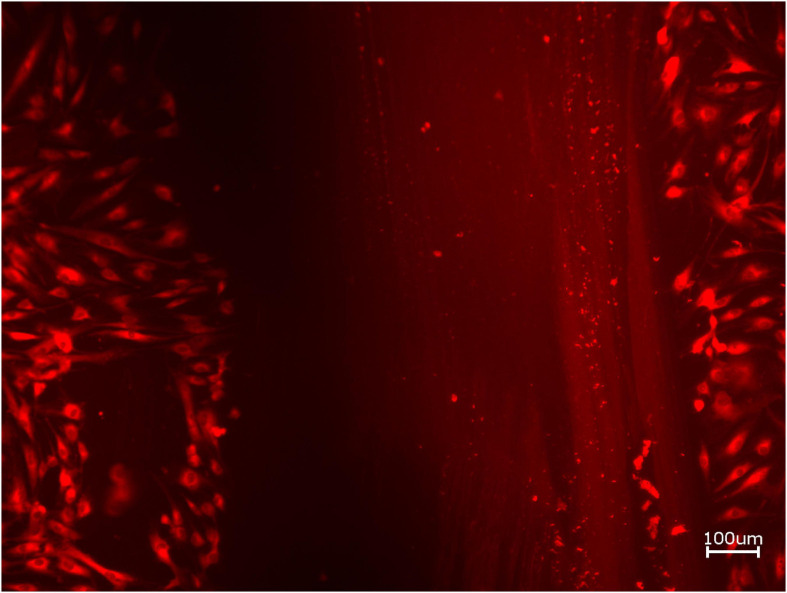
Exemplary micrographs of migrated HOBs assessed via scratch test assay for allogeneic BSM without the addition of PRF (10× magnification).

**FIGURE 6:**
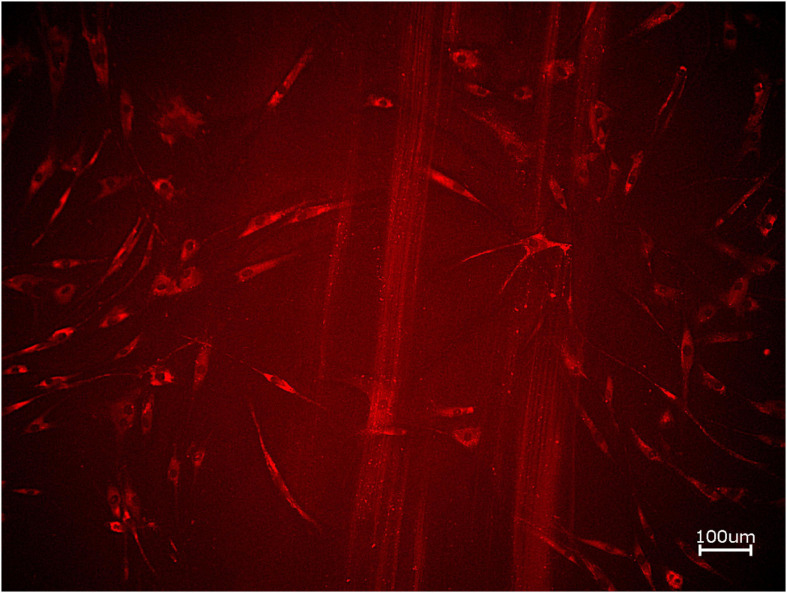
Exemplary micrographs of migrated HOBs assessed via scratch test assay for allogeneic BSM with the addition of PRF (10× magnification).

**FIGURE 7:**
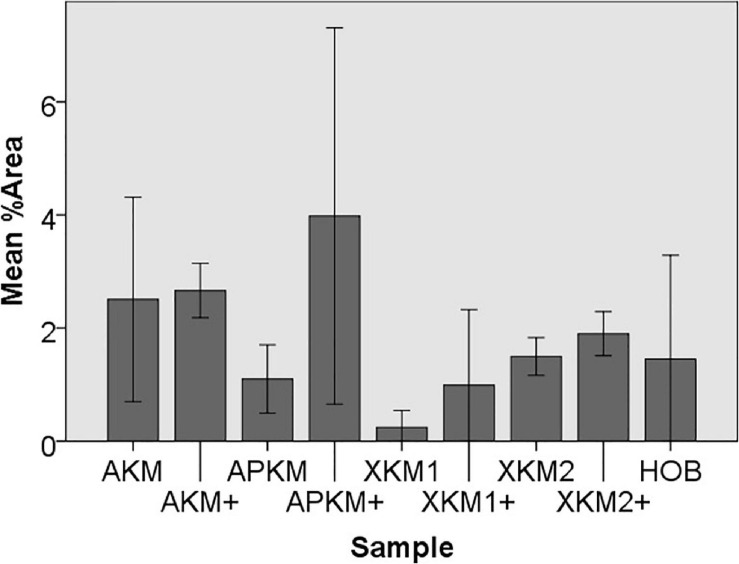
Mean percentage of migrated cells assessed via scratch assay of HOB after co-incubation with the respective samples with (+)/without PRF (each *p* > 0.05, Mann–Whitney *U* testing in comparison to HOB and native BSM).

To assess the differences between the groups concerning cell migration, the scratch assay was assessed (three samples in duplets for each, *n = 54*, Figures 5–7). Here, comparisons between all samples did not reveal any statistically significant differences (p = *0.467).* However, the percentage of HOB migrated into the gap after 24 h was slightly higher for all groups when PRF was added and was the highest for APKM (almost doubled in comparison to APKM alone) but failed to show statistical significance when compared to HOB alone (all tested samples: p *> 0.05*). In comparison to their native BSM, PRF tended *to increase cell migration* for alloplastic material but no statistical significance differences where found (all tested samples: p *> 0.05*, Table 2C).

### PRF in Combination With Different BSM Triggers Early Release of Marker for Osteoblast Proliferation and Differentiation

To further characterize the early interaction of PRF with the respective BSM and their influence on osteoblasts, the evident marker of proliferation and differentiation on gene and protein level via PCR and ELISA quantification, respectively, were analyzed.

#### Gene Expression

**FIGURE 8:**
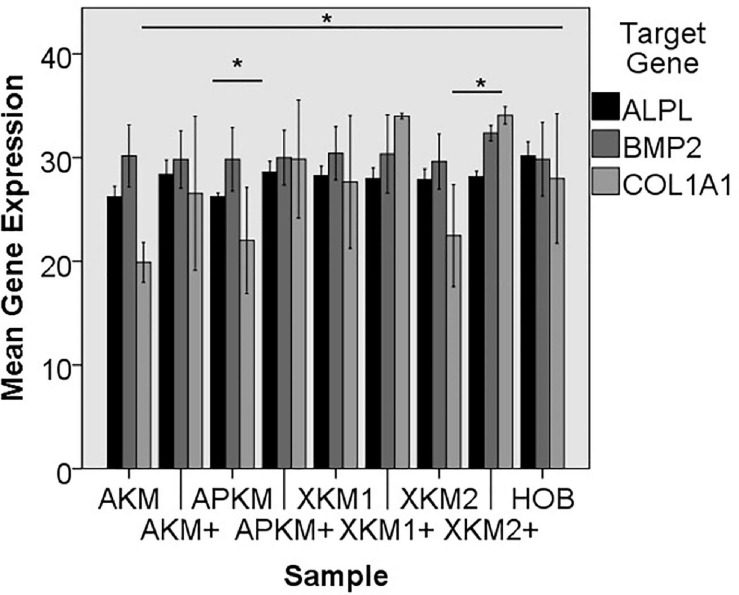
Mean gene expression of *ALPL, BMP2, and COL1A1* after co-incubation of HOB with the respective samples with (+)/without PRF (*p < 0.05, Mann–Whitney *U* testing in comparison to HOB and native BSM, *ALPL:* APKM vs. APKM+: *p* = 0.050, *COL1A1*:AKM vs. HOB: *p* = 0.050, XKM2 vs. XKM2+: *p* = 0.050).

**FIGURE 9:**
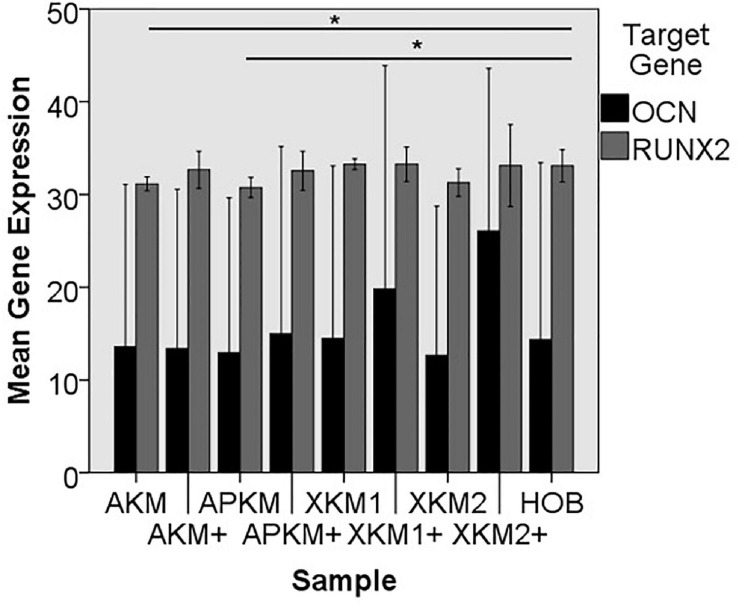
Mean gene expression of *OCN* and *RUNX2* after co-incubation of HOB with the respective samples with (+)/without PRF (*p < 0.05, Mann–Whitney *U* testing in comparison to HOB and native BSM, *RUNX2:* AKM vs. HOB: *p* = 0.050, APKM vs. HOB: *p* = 0.050).

The PCR results (three samples in triplets each per gene, *n* = 36, Figures 8, 9) showed no significant differences between the groups (*p* = 0.069, Tables 3A–E).

**TABLE 3:** Gene expression of **(A)** *ALPL*, **(B)** *BMP2*, **(C)** *COL1A1*, **(D)** *OCN*, **(E)** *RUNX2* assessed via PCR for the respective samples and respective *p*-values vs. HOB alone and native BSM.

(A)			
**Sample**	**Mean *ALPL* expression**	***p*-Value (respective sample vs. HOB, Mann–Whitney *U* test)**	***p*-Value (respective sample with PRF vs. native control, Mann–Whitney *U* test)**

HOB	30.14 ± 1.37	–	–
AKM	26.02 ± 1.02	0.083	0.127
AKM+	28.382 ± 1.37	0.248	
APKM	26.234 ± 0.350	0.083	0.050
APKM+	28.5595 ± 1.05	0.248	
XKM1	28.266 ± 0.902	0.121	0.564
XKM1+	27.963 ± 1.039	0.439	
XKM2	27.859 ± 1,04	0.083	0.827
XKM2+	28.147 ± 0.54	0.083	

**(B)**			

**Sample**	**Mean *BMP2* expression**	***p*-Value (respective sample vs. HOB, Mann–Whitney *U* test)**	***p*-Value (respective sample with PRF vs. native control, Mann–Whitney *U* test)**

HOB	29.837 ± 3.572	–	–
AKM	30.151 ± 2.992	0.827	0.827
AKM+	29.813 ± 2.77	0.827	
APKM	29.834 ± 3.065	0.827	0.827
APKM+	29.998 ± 2.642	0.827	
XKM1	30.420 ± 2.57	0.827	1.00
XKM1+	30.336 ± 3.780	1.00	
XKM2	29.623 ± 2.651	0.827	0.127
XKM2+	32.356 ± 0.747	0.513	

**(C)**			

**Sample**	**Mean *COL1A1* expression**	***p*-Value (respective sample vs. HOB, Mann–Whitney *U* test)**	***p*-Value (respective sample with PRF vs. native control, Mann–Whitney *U* test)**

HOB	27.981 ± 6.247	–	–
AKM	19.897 ± 1.918	0.050	0.127
AKM+	26.546 ± 7.4264	0.827	
APKM	22.0 ± 5.1184	0.275	0.127
APKM+	29.85 ± 5.688	0.513	
XKM1	27.641 ± 6.406	0.513	0.564
XKM1+	33.996 ± 0.280	0.564	
XKM2	22.466 ± 4.931	0.275	0.050
XKM2+	34.064 ± 0.841	0.513	

**(D)**			

**Sample**	**Mean *OCN* expression**	***p*-Value (respective sample vs. HOB, Mann–Whitney *U* test)**	***p*-Value (respective sample with PRF vs. native control, Mann–Whitney *U* test)**

HOB	14.340 ± 19.083	–	–
AKM	13.595 ± 17.499	0.827	0.827
AKM+	13.3773 ± 17.186	0.827	
APKM	12.965 ± 16.68	0.827	0.827
APKM+	14.988 ± 20.209	0.827	
XKM1	14.474 ± 18.596	0.827	0.564
XKM1+	19.809 ± 24.069	0.564	
XKM2	12.68 ± 16.060	0.827	0.127
XKM2+	26.05 ± 17.529	0.275	

**(E)**			

**Sample**	**Mean *RUNX2* expression**	***p*-Value (respective sample vs. HOB, Mann–Whitney *U* test)**	***p*-Value (respective sample with PRF vs. native control, Mann–Whitney *U* test)**

HOB	33.095 ± 1.7340	–	–
AKM	31.143 ± 0.744	0.050	0.513
AKM+	32.668 ± 2.008	0.827	
APKM	30.758 ± 1.082	0.050	0.257
APKM+	32.556 ± 2.109	0.275	
XKM1	33.265 ± 0.599	0.513	1.00
XKM1+	33.273 ± 1.859	0.564	
XKM2	31.285 ± 1.488	0.275	0.513
XKM2+	33.113 ± 4.418	0.827	

The *ALPL* expression was highest for HOB alone in comparison to other samples (all tested samples: *p* > 0.05). In comparison to the native BSM, the mean expression for *ALPL* was higher for each BSM when PRF was added with a significant increase for alloplastic material (APKM vs. APKM+: *p* = 0.050, all other tested samples: *p* > 0.05). *BMP2* gene expression tended to be increased for all the tested samples in comparison to HOB alone (all tested samples: *p* > 0.05) and for the combination of PRF and the respective material in comparison to the native BSM (all tested samples: *p* > 0.05). Allogeneic BSM significantly decreased the *COL1A1* expression in comparison to HOB alone (*p* = 0.050), but other samples did not (all other tested samples: *p* > 0.05). In comparison to the native BSM, the *COL1A1* expression was significantly increased for PRF in combination with the combination of xenogeneic material 2 with PRF (*p* = 0.050, all other tested samples: *p* > 0.05). For *OCN* expression, no significant difference for the tested material in comparison to HOB alone (all tested samples: *p* > 0.05) and between bio-activated and native BSM (all tested samples: *p* > 0.05) was found. Allogeneic and alloplastic BSM displayed a significant increase in the *RUNX2* expression, whereas the other analyzed BSM did not show noteworthy differences (AKM: *p* = 0.050, APKM: *p* = 0.050, all other tested samples: *p* > 0.05). Combination of the respective sample with PRF did not significantly increase the RUNX-2 expression vs. the native BSM (all tested samples: *p* > 0.05).

#### Protein Expression

**FIGURE 10:**
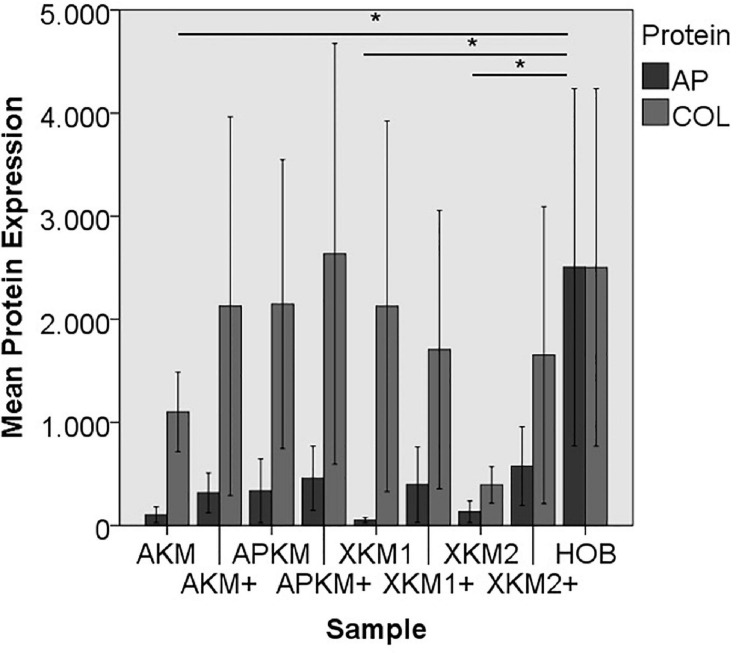
Mean protein expression of AP and COL after co-incubation of HOB with the respective samples with (+)/without PRF (*p < 0.05, Mann–Whitney *U* testing in comparison to HOB and native BSM, AP: AKM vs. HOB: *p* = 0.050, XKM1 vs. HOB: *p* = 0.050, XKM2 vs. HOB: *p* = 0.050).

**FIGURE 11:**
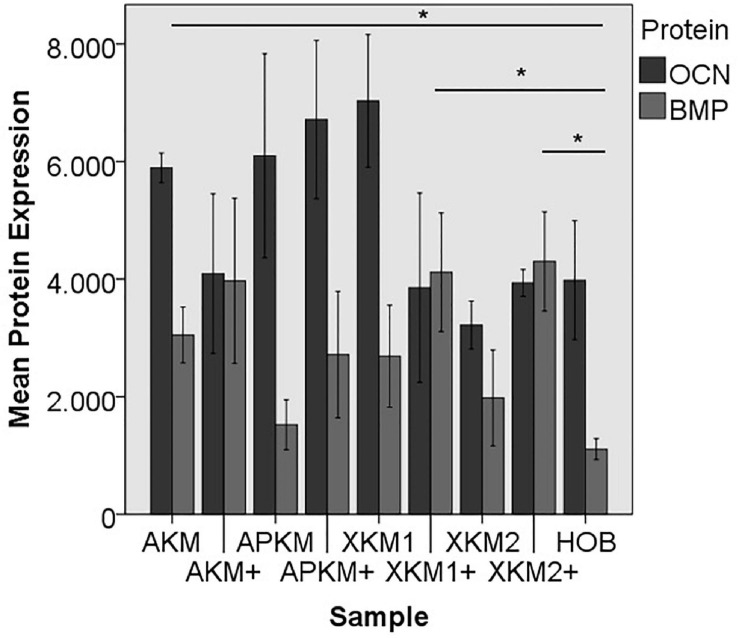
Mean protein expression of OCN and BMP after co-incubation of HOB with the respective samples with (+)/without PRF (*p < 0.05, BMP: AKM vs. HOB: *p* = 0.050, XKM1+ vs. HOB: *p* = 0.050, XKM2+ vs. HOB: *p* = 0.050).

Next, ELISA quantification (five samples in triplets each per antibody, *n* = 60, Figures 10, 11) was done to analyze the differences on protein basis. There was no significant difference between all the tested samples (p = 0.069, Tables 4A–D).

**TABLE 4:** textbf(A) AP, **(B)** COL, **(C)** OCN, **(D)** BMP protein expression assessed via ELISA for the respective samples and respective *p*-values vs. HOB alone and native BSM.

(A)			
**Sample**	**Mean AP protein expression**	***p*-Value (respective sample vs. HOB, Mann–Whitney *U* test)**	***p*-Value (respective sample with PRF vs. native control, Mann–Whitney *U* test)**

HOB	3980.53 ± 1751.86	–	–
AKM	5893.47 ± 437.749	0.050	0.275
AKM+	4091.66 ± 2352.30	0.127	
APKM	6099.42 ± 3006.82	0.127	0.275
APKM+	6713.18 ± 2330.33	0.127	
XKM1	7031.43 ± 1955.22	0.050	0.275
XKM1+	3855.01 ± 2789.08	0.127	
XKM2	3219.49 ± 706.21	0.050	0.275
XKM2+	3935.84 ± 392.31	0.275	

**(B)**			

**Sample**	**Mean COL protein expression**	***p*-Value (respective sample vs. HOB, Mann–Whitney *U* test)**	***p*-Value (respective sample with PRF vs. native control, Mann–Whitney *U* test)**

HOB	2502.06 ± 3004.99	–	–
AKM	1101.07 ± 666.66	0.827	0.513
AKM+	2126.60 ± 3181.29	0.275	
APKM	2146.64 ± 2427.82	0.827	0.127
APKM+	2634.02 ± 3533.55	0.827	
XKM1	2124.46 ± 3113.57	0.513	0.275
XKM1+	1705.80 ± 2339.48	0.439	
XKM2	394.48 ± 305.84	0.127	0.439
XKM2+	1652.37 ± 2495.20	0.275	

**(C)**			

**Sample**	**Mean OCN protein expression**	***p*-Value (respective sample vs. HOB, Mann–Whitney *U* test)**	***p*-Value (respective sample with PRF vs. native control, Mann–Whitney *U* test)**

HOB	1107.52 ± 310.94	–	–
AKM	3048.80 ± 824.21	0.127	0.513
AKM+	3971.44 ± 2436.41	0.827	
APKM	1524.62 ± 734.12	0.275	0.827
APKM+	2715.51 ± 1862.22	0.127	
XKM1	2689.14 ± 1504.79	0.127	0.275
XKM1+	4117.33 ± 1746.73	0.827	
XKM2	1977.93 ± 1412.79	0.513	0.127
XKM2+	4300.08 ± 1460.49	0.513	

**(D)**			

**Sample**	**Mean BMP2 protein expression**	***p*-Value (respective sample vs. HOB, Mann–Whitney *U* test)**	***p*-Value (respective sample with PRF vs. native control, Mann–Whitney *U* test)**

HOB	2503.98 ± 3001.88	–	–
AKM	103.56 ± 132.29	0.050	0.513
AKM+	315.06 ± 336.14	0.127	
APKM	336.50 ± 536.87	0.513	0.275
APKM+	456.91 ± 540.82	0.275	
XKM1	50.21 ± 42.82	0.127	0.275
XKM1+	396.22 ± 634.17	0.050	
XKM2	132.69 ± 181.62	0.275	0.275
XKM2+	575.36 ± 661.23	0.050	

ALP expression was found to be highest for HOB alone with a significant decrease for allogeneic and xenogeneic samples (AKM: *p* = 0.050, XKM1: *p* = 0.050, XKM2: *p* = 0.050, all other samples: *p* > 0.05). Furthermore, all BSM in combination with PRF tended to increase ALP expression (all tested samples: *p* > 0.05). For COL expression, there were no statistical significant differences of the respective samples in comparison to HOB alone (all tested samples: *p* > 0.05) and the native BSM (all tested samples: *p* > 0.05). Similarly, OCN expression did not have a significant statistical difference in comparison to HOB alone (all tested samples: *p* > 0.05) and combination of PRF and the respective BSM vs. native material (all tested samples: *p* > 0.05). BMP expression was increased for allogeneic (*p* = 0.050) and the combination of PRF and xenogeneic materials in comparison to HOB alone (XKM1+: *p* = 0.050, XKM2+: *p* = 0.050). Furthermore, PRF addition tended to increase BMP expression for the respective BSM vs. native material, however without reaching statistical significance (all tested samples: *p* > 0.05).

## Discussion

Within this study, a comparative analysis of the initial interaction of the combination of different BSM with PRF and its possible influence on early osteoblast viability, proliferation, and migration were performed *in vitro.*

As a major result, the combination of PRF with different BSM increases initial viability of HOBs. Furthermore, marker of proliferation and differentiation on gene and protein level, especially *RUNX2*, alkaline phosphatase, and collagen-1 demonstrated a noteworthy increase after co-incubation with BSM in addition to PRF and HOB in comparison to HOB alone for 24 h.

Other *in vitro* studies demonstrate ambivalent results where PRF did not significantly affect the expression of osteoblastic marker genes for differentiation, encoding ALP, RUNX2, or BMP2 (Sumida et al., 2019). Here, ALP mRNA levels were even decreased in comparison to premature osteoblasts alone. As a possible explanation, the authors state that ALP activity is high in mature osteoblasts and PRF did not inhibit, but rather delay the peak of osteoblast differentiation. This regulation may optimize bone remodeling to an osteogenic state during the early osteoblastic differentiation stages before ALP expression gradually increased over time (Sumida et al., 2019). This is in line with the presented results, where PRF led to an increase of the ALPL gene expression after 24 h. In addition, other studies found that TGF-β and PDGF, both growth factors released by PRF, may even reduce alkaline phosphatase and consequently delay differentiation (Strauss et al., 2020). Therefore, it can be discussed if PRF predominately assists in early stage osteogenesis by optimizing primarily osteoblast differentiation (Sumida et al., 2019). The increased collagen expression found in this study is also in accordance with the literature where other *in vitro* studies proved that PRF increased osteoblast attachment and proliferation via upregulating collagen-related protein production (Wu et al., 2012). Furthermore, the elevated BMP and *RUNX2* expressions in the combination of PRF especially with allogeneic BSM may additionally induce osteoprotegerin and promote bone forming activity by increased collagen or osteocalcin production (Engler-Pinto et al., 2019; Sumida et al., 2019).

This is seen in the presented significant increased cell viability via MTT assay especially for xenogeneic BSM. In a recent analysis, the negative effect of zoledronic acid on the viability and proliferation of osteoblasts could partly be reversed by the application of PRF (Steller et al., 2019). In this study, differentiation and proliferation of osteoblasts tended to be increased when BSMs were combined with PRF but failed to show significant differences. Here, further immunological features should be addressed in subsequent studies to understand the cellular background. Using a first generation PC (Platelet Rich Plasma, PRP), the combination of PC and carbonated hydroxyapatite tended to decrease pro-inflammatory cell inflammation and subsequently showed a histologically increased bone formation (Oley et al., 2018).

This study suffers from some limitations. First and foremost, *in vitro* studies lack the general bias that results cannot reflect complex interactions in a biological system that may distort the effects. However, only *in vitro* analysis allows drawing conclusions about single cell-cell interaction. Next and in accordance with the literature, only one human osteoblast cell line was used for analysis. Surely, a multi cell line approach could strengthen the discussed hypothesis and should therefore be included in future studies. Additionally, this study solemnly focuses on the initial and early interaction of PRF and BSM and implications for HOB’s viability proliferation and differentiation. This way, new insights in the underlying intercellular processes and protein release kinetics may be gained in comparison to the complemented data in the literature. However, subsequent time points are not validated in this analysis. Finally, most of the given results did not reach statistical significance. However, since only small sample sizes (as a further limitation) were analyzed, statistical significance should be treated with caution and may reflect overall limited validity. Taken together, future *in vivo* studies are much in need to validate the found tendencies.

Within the named limitation of the presented approach, no recommendation can be given which BSM may best optimize bony regeneration in combination with PRF. However, without reaching statistical significance, alloplastic and especially xenogeneic BSM interacted strongly with PRF and did influence osteoblast features the most.

The possible underlying intercellular mechanism and early angiogenic interactions of the PRF with the respective BSM were evaluated in another study by our working group (Blatt et al., 2021). Here, it was demonstrated that PRF initially interacts with its respective BSM via platelet activation *in vitro*. Furthermore, PRF had a significant positive pro-angiogenic effect, especially in combination with alloplastic and xenogeneic materials *in vivo*. Here, validated by scanning electron microscopy, a “storage” of the respective growth factors of the PRF via the close spatial relationship between the fibrin network and the BSM and a consecutive slow release that triggers vasoformative responses was hypothesized (Blatt et al., 2021). This assumption may be transferred to the implications of bony regeneration and could explain the release kinetics and expression of the above investigated markers found in this study: initially, PRF boosts primary viability of HOBs and subsequently releases differentiation and migration marker. This hypothesis also explains the fact that migration assay did demonstrate a noteworthy influence of the PRF but failed to reach statistical significance at this early time point.

This postulation is validated by another recent analysis by Kyyak et al. (2020) that investigated if the combination of an allogeneic or a xenogeneic BSM in combination with PRF may influence osteoblast activity after longer incubation time points (after 3, 7, and 14 days). It was shown that the addition of PRF to allogeneic and, to a minor content, to xenogeneic BSM revealed a significant increase of HOB viability, migration, proliferation, and differentiation (Kyyak et al., 2020). In a bone remodeling animal study, the incorporation of PRF into a carbonated hydroxyapatite loaded hydrogel demonstrated a higher number of osteoblasts and decreased osteoclast activity in comparison to BSM alone after 14 and 21 days (Alhasyimi et al., 2018). Therefore, it can be discussed if the combination of PRF and BSM predominately optimizes early stage osteogenesis whereas a significantly increased expression is seen at later time points after the passive release of the growth factors physically entrapped within the fibrin network. At this point in time, allogeneic BSMs that seem to bear osteoconductive properties may be in favor to increase angiogenesis and new vessel sprouting (Kyyak et al., 2020). In context with the above-mentioned hypothesis, future studies should investigate if biomechanical aspects of the investigated BSM may influence interactions with PRF to a greater extent than what was previously assumed. This may broaden the indications of bioceramics and other BSM in regenerative medicine (Ana et al., 2018).

## Conclusion

To conclude, PRF in combination with different BSM led to a noteworthy early influence on osteoblast proliferation, differentiation, and viability *in vitro*. In contrast to other bone-engineering methods that are hardly integrated in clinical workflow (mostly due to regulatory and practically restrictions), PC and especially PRF are autologous materials that are easy to produce and use chair-side. As shown, they seem capable to enhance the features that optimize bony regeneration. Therefore, translation in the clinical pathway seems feasible.

## Data Availability Statement

The original contributions presented in the study are included in the article/supplementary material, further inquiries can be directed to the corresponding author.

## Ethics Statement

The studies involving human participants were reviewed and approved by Ärztekammer Rheinland-Pfalz, vote no. “2019-14705_1.” The patients/participants provided their written informed consent to participate in this study.

## Author Contributions

SB and AP contributed to the conceptualization. PK and SK contributed to the methodology. SB, DT, and AP contributed to the validation. BA-N and PK contributed to the formal analysis and supervision. SB, DT, and AP contributed to the investigation. PK and SK contributed to the data curation. SB and PK contributed to the writing—original draft preparation. DT, AP, and BA-N contributed to the writing—review and editing. SK contributed to the visualization. AP contributed to the project administration. PK contributed to the funding acquisition. All authors have read and agreed to the published version of the manuscript.

## Conflict of Interest

The authors declare that the research was conducted in the absence of any commercial or financial relationships that could be construed as a potential conflict of interest.
